# Supplementary material for: Pain Prevalence and Satisfaction with Pain Management in Inpatients: A Cross-Sectional Study
Source: Healthcare (Basel). 2023 Dec 18;11(24):3191. doi: 10.3390/healthcare11243191 (PMC10743299; doi:10.3390/healthcare11243191)
Supplement: Supplementary file 1 [file healthcare-11-03191-s001.zip › File S1.pdf]

Pain prevalence in hospitalized patients and satisfaction regarding pain management

Questionnaire - NCT04901650 (English translation)

Age: ___ years old

Gender:
- o Male
- o Female

Marital Status:
- o Single
- o In a relationship/Married
- o Divorced/Widowed

Race:
- o Caucasian
- o Ethiopian
- o Asian

Religion:
- o Atheist
- o Christian
- o Muslim
- o Protestant
- o Others

Occupation:
- o Employed
- o Retired
- o Housework
- o Unemployed

Comorbidities: ____

____

____

____

____

____

____

Service in charge of patient care:
- o Surgical
- o Non-Surgical

Type of hospital admission:
- o Emergent
- o Scheduled

Length of hospital stay: ________ days

Do you currently experience any type of pain?

- Yes
- No

Indicate the intensity of your current pain:

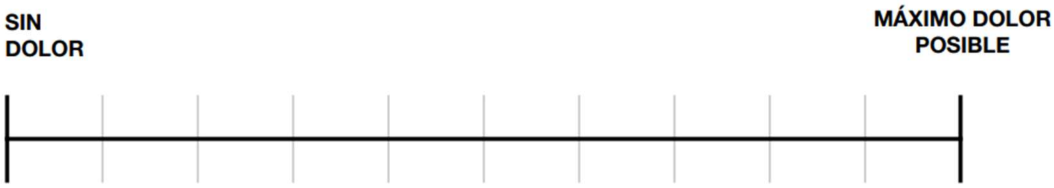

Please mark on the diagram below the area of the body where your pain is located. If you have more than one area of pain, mark the area where pain is of greatest intensity:

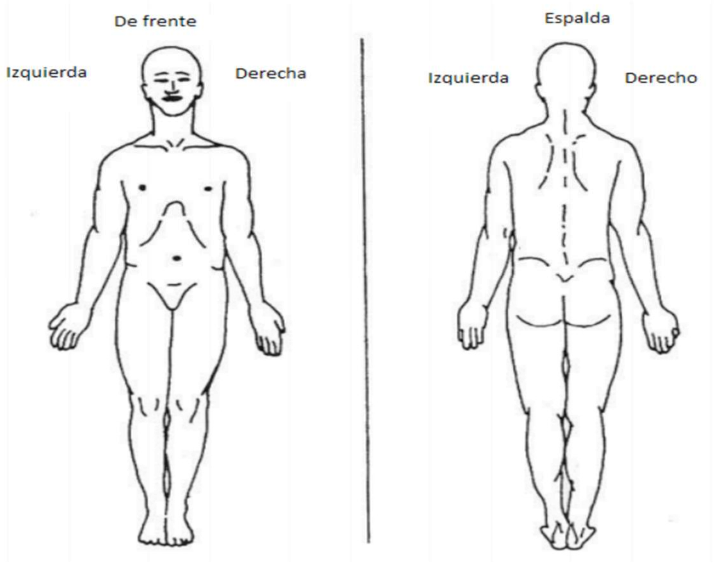

Please indicate from 0 to 10 how satisfied you are with the hospital's pain management

(0 = not satisfied, 10 = maximum satisfaction)

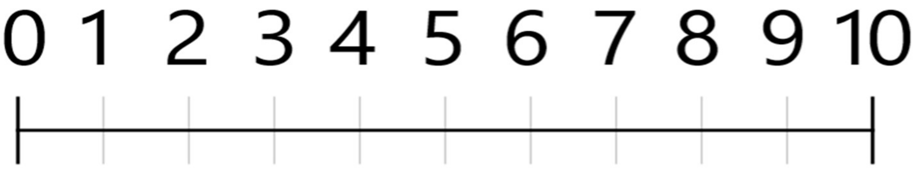

Compared with pain experienced prior to hospital admission, the pain you suffer has:

- Decreased
- Not changed
- Worsened

Treatment for pain in electronic medical records:

- Yes:____
- No
